# Associations between Domestic-Dog Morphology and Behaviour Scores in the Dog Mentality Assessment

**DOI:** 10.1371/journal.pone.0149403

**Published:** 2016-02-26

**Authors:** Holly R. Stone, Paul D. McGreevy, Melissa J. Starling, Bjorn Forkman

**Affiliations:** 1 Faculty of Veterinary Science, University of Sydney, Sydney, New South Wales, 2006, Australia; 2 University of Copenhagen, Copenhagen, Denmark; Faculty of Animal Sciences and Food Engineering, University of São Paulo, BRAZIL

## Abstract

The domestic dog shows a wide range of morphologies, that humans have selected for in the process of creating unique breeds. Recent studies have revealed correlations between changes in morphology and behaviour as reported by owners. For example, as height and weight decrease, many undesirable behaviours (non-social fear, hyperactivity and attention seeking) become more apparent. The current study aimed to explore more of these correlations, but this time used reports from trained observers. Phenotypic measurements were recorded from a range of common dog breeds (n = 45) and included cephalic index (CI: the ratio of skull width to skull length), bodyweight, height and sex. These data were then correlated with results from the Dog Mentality Assessment (DMA), which involves trained observers scoring a dog’s reaction to stimuli presented over 10 standardised subtests. Each subtest is designed to evoke a behavioural response. Backward elimination and weighted step-wise regression revealed that shorter dogs demonstrated more aggressive tendencies, reacting defensively toward both assistants dressed as ghosts (p = 0.045), and to a dummy (p = 0.008). Taller dogs were more affectionate when greeting and being handled by humans (p = 0.007, p = <0.001, respectively). Taller dogs were also more cooperative (p = <0.001), and playful (p = 0.001) with humans than shorter dogs. Heavier dogs were more inquisitive toward a dummy (p = 0.011), to the source of a metallic noise (p = 0.010) and to an assistant (p = 0.003). Heavier dogs were also more attentive to the ghosts (p = 0.013). In comparison, lighter dogs were cautious of a dummy (p = <0.001) and fearful of the sound of a gunshot (p = <0.001). Lighter dogs were also cautious of, and demonstrated prolonged fearfulness toward, the source of metallic noise (p = <0.001, p = <0.034, respectively). With a far larger sample and the advantage of third-party reporting (which overcomes potential owner bias), the current findings build on previous studies in this field, further supporting covariance between morphology and behaviour.

## Introduction

Throughout the process of domestication, the dog has been transformed to meet many of the functional and emotional needs of humans [1]. Selective breeding has produced a broad spectrum of dogs occupying various niches as herders, hunters, protectors, trackers, assistance dogs, athletes and, commonly, as companions. Their relationships with humans, their ongoing journey to domestication and their morphological diversity have captured the interest of geneticists, anatomists and ethologists. Since the introduction of dog shows and the emergence of the Kennel Club in the UK 141 years ago [2]. breeding has become more formal. Written breed standards have provided a blueprint for the morphological characteristics that have been selected by breeders for conformation classes [3].

There are more than 400-recorded breeds [4], each with its own characteristic morphology [5–6]. Skull morphology, in particular, varies dramatically among breeds. For example, skull length varies from 7cm to 28cm in length [7–8]. Retzius (1840) provided the first classifications of human cranial morphology, specifically dolichocephalic and brachycephalic [9]. Mesaticephalic was recognised later. In broad terms, dolichocephalic is a long and narrow head, mesaticephalic is a head of medium proportions and brachycephalic is a head of short and wide proportions [10].

Behaviourally, dolichocephalic breeds excel as hunting dogs. Their success could relate to their skull shape, which is associated with a distribution of retinal ganglion cells that gives them an enhanced ability to scan the peripheral visual field when chasing prey [7, 11]. In contrast, brachycephalic breeds have long been popular as so-called lap dogs. Their foreshortened skull shape is associated with eyes that are more forward-facing and that have greater central visual acuity than that of dogs with longer skulls [7]. McGreevy et al. (2003) suggested that brachycephalic dogs may have a reduced ability to detect horizontal motion, which, in turn, may make them less likely than dogs with longer skulls to show hunting behaviours [12]. Recent research has also indicated that features commonly associated with brachycephalic dogs (e.g., a relatively longer distance between the eyes), have been shown to be more appealing to humans [13]. These craniofacial features and behavioural differences may give rise to the impression that brachycephalic dogs are more engaged with their owners [7].

While dolichocephalic, mesaticephalic and brachycephalic are useful for classification of dog skulls, the terms have been criticised as being overly simplistic [4, 14]. The use of cephalic index (CI) to capture the ratio between maximum width and maximum length of the skull provide an estimate of cranial morphology in a continuum across all skull shapes and sizes [3, 8]. However, Georgevsky et al. (2014) report that CI may not be sensitive enough to capture some subtle differences in head shape, especially in smaller dogs [14]. Dogs with significantly different head shapes, such as the Papillon and the Pug, can have a very similar CI calculation [14]. CI is measured on live dogs, so exact measurements of the skull are hindered by the presence of living tissue covering the head [14].

In addition to the range of skull sizes, there is enormous variety in height and bodyweight within breeds and in some cases between sexes within a breed. Chihuahuas are at the small end of the spectrum, measuring only 20cm and 2kg, compared to Newfoundlands that can reach 70cm and 60kg [5]. Studies have shown an association with body size and behaviour. The larger the dog, the less likely it is to display anxiety, fear or neuroticism [15]. Smaller dogs also behave more aggressively than larger dogs, by barking, growling, baring teeth, snapping, lunging, and biting or attempting to bite humans [16].

Recently, McGreevy et al. (2013) have shown that a dog’s CI, bodyweight and height all correlate with a dog’s behavioural characteristics [17]. In this study, behavioural data were drawn from the Canine Behavioural and Research Questionnaire (C-BARQ), an online questionnaire completed by owners. McGreevy et al. (2013) showed that 32 undesirable behaviours were associated with either height alone, bodyweight alone, CI alone, bodyweight and CI combined, height and CI combined or height and bodyweight combined [17]. Such undesirable behaviours included mounting persons or objects, begging for food and separation-related problems. Shorter dogs demonstrated more attention seeking and fearful behaviours than taller dogs [17]. Supporting previous research, this study found that shorter dogs also demonstrated more aggression, toward both strangers and owners [17]. As bodyweight decreases, excitability and hyperactivity increase [17]. Interestingly, stereotypic behaviours, such as tail-chasing, correlated negatively with height but positively with weight [17]. These short, stocky dogs also demonstrated other behaviours that may be indicative of distress, such as emotional urination [17]. It is not understood why dogs of this morphology are more likely to need to adopt a cluster of so-called coping behaviours [17].

McGreevy et al. (2013) considered possible explanations for their finding that as height and weight decrease, unwelcome behaviours become more apparent [17]. It is possible that as a smaller body size is selected for, the neurological anatomy may also be genetically altered. Neurological changes could be responsible for the expression of certain innate behaviours, such as reactivity [17]. Smaller dogs may be innately more reactive than larger dogs to the same stimuli [17]. The environment in which a dog lives may, in part at least, reflect its size. Bigger dogs may be housed outdoors more often than smaller dogs [17]. Undesirable behaviours may be better tolerated by small dogs in a household than the same behaviour by a large dog would be [17]. As a result, more emphasis may have been placed on breeding for acceptable temperaments in large breeds, while this may not be so critical in smaller breeds [17]. Small dogs with a high CI (i.e., brachycephalic dogs) may have been selected for a neotenous appearance [13]. At the same time, it is possible that selection pressure has been applied in smaller breeds to actually favour neotenous behaviours, such as attachment and fearfulness [17]. So smaller dogs may be managed in ways that compromise their ability to express normal behaviours and thus may increase frustration and trigger the emergence of unwelcome behaviours. There are many potential explanations for the correlations between morphology and behaviour that require further investigation.

The current study continues to examine these correlations using data from the Dog Mentality Assessment (DMA). The DMA is used by the Swedish Working Dog Association (SWDA) to test behavioural reactions of dogs to standardised stimuli [18]. The primary purpose of the DMA was to identify desirable and undesirable behavioural characteristics in the sire or dam prior to breeding [18]. This information could then be considered when calculating estimated breeding values. The DMA was initially used only for working breeds, but is now open to other breeds and can be considered a broad behavioural assessment [18]. At set time periods throughout the assessment, trained observers score the dog’s behaviour in a standardised manner. This scoring system allowed reliable comparisons to be made between breeds for this study. The different subtests of the DMA have been shown to have good to fair repeatability over time, so the behavioural response of the dog reflects a stable personality trait [19].

Uncovering links between phenotypic variables and aspects of a dog’s behaviour, such as playfulness, fearfulness, aggression or sociability, could have profound implications. We may discover that behaviours undesirable in a companion animal have been inadvertently selected for when breeding to achieve a specific phenotype. We may also reveal that certain physical parameters are worth selecting when planning future progeny destined for a particular working role, such as drug-detection or assistance dogs.

## Materials and Methods

### Cephalic Index

A representative sample of dogs (n = 12; 6 females and 6 males) from each breed (n = 45) was measured to provide an average CI per breed. The breeds selected were recognised by the Australian National Kennel Council (ANKC) and had a minimum of 30 annual registrations. This was to eliminate rare breeds from the study.

Dogs could be included only if they were of show quality or were from show-quality lines. The dog had to be at least two years of age and could not be a littermate of any dogs already participating in the study. The owner was required to be a breeder registered with Dogs NSW, and had to have registered at least 30 puppies nationally with the ANKC in 2009. Owners gave permission for their dogs to be used in the study.

Each dog’s head was held with the nasal planum in a horizontal position and a standardised cloth strap, with a rectangular benchmark of 2.5cm x 4.9cm, was placed around the widest part of the skull. The assistant placed a finger on the occipital crest and a photograph was then taken providing a dorsoventral view of the head. Each dog’s breed, name and age were recorded. Once the photographs were collected, a GNU image manipulation program GIMP 2.8.0 (http://www.gimp.org/) was used. After normalisation to the reference rectangle, the width of the skull was calculated. The length of the skull was determined by measuring the distance between the occipital crest and the most anterior point of the nose. Cephalic index was calculated as 100 x skull width divided by the skull length.

The University of New South Wales Animal Ethics Committee approved this data collection as it was non-invasive. Show dogs are generally used to being approached by judges and examined while being held by their owners. The dogs in the current series exhibited no signs of distress in response to this sampling method.

### Height and Bodyweight

Data on the preferred heights and bodyweights for each breed were recorded from a dog information portal (www.dogbreedinfo.com). Where a range was listed, the median was calculated. If the preferred height or bodyweight differed for the male and female within a breed, the mean was calculated. If www.dogbreedinfo.com did not list a preferred height or weight for a breed, data was instead obtained from another dog information portal (www.purina.com.au). Only two breeds did not have a preferred height or weight listed on www.dogbreedinfo.com –the Chinese Crested dog and Finnish Lapphund.

### Dog Mentality Assessment

The subjects used were 67,368 dogs from 45 breeds (Table 1). There were 32,790 males and 34,578 females. All dogs were companion animals. The average age at the time of assessment was 637 days.

**Table 1:** Summary of the number and gender of 67,368 dogs from 45 breeds from which the DMA data were sourced.

Breed	Females	Males	Total
American Staffordshire Terrier	410	358	768
Australian Cattle Dog	158	116	274
Australian Kelpie	752	704	1456
Australian Shepherd	1083	1065	2148
Australian Terrier	23	28	51
Bearded Collie	102	115	217
Bernese Mountain Dog	972	884	1856
Border Collie	369	500	869
Boston Terrier	36	15	51
Boxer	2594	2598	5192
Bull Terrier	138	107	245
Bullmastiff	33	28	61
Cairn Terrier	96	108	204
Cavalier King Charles Spaniel	45	46	91
Chinese Crested Dog	28	31	59
Cocker Spaniel	191	196	387
Collie (Rough)	1664	1562	3226
Dalmatian	226	191	417
Doberman	1149	1150	2299
English Springer Spaniel	481	460	941
Finnish Lapphund	151	182	333
French Bulldog	122	84	206
German Pinscher	257	268	525
German Shepherd	10601	9711	20312
German Shorthaired Pointer	130	148	278
Golden Retriever	1878	2125	4003
Gordon Setter	33	36	69
Great Dane	53	73	126
Irish Setter	58	67	125
Jack Russell Terrier	190	181	371
Labrador Retriever	1464	1398	2862
Lagotto Romagnolo	142	159	301
Leonberger	663	548	1211
Papillon	53	68	121
Pug	25	20	45
Rhodesian Ridgeback	1043	1053	2096
Rottweiler	5757	5031	10788
Samoyed	48	61	109
Schnauzer (Miniature)	435	446	881
Shetland Sheepdog	149	160	309
Staffordshire Bull Terrier	576	498	1074
Tibetan Terrier	38	35	73
Welsh Corgi (Cardigan)	46	49	95
West Highland White Terrier	31	24	55
Whippet	85	103	188

The data were collected between 1997 and 2014, at 235 testing arenas in Sweden by 307 official observers, who had been trained to score a dog’s reaction according to pre-set standards, with an emphasis on recording an objective description of the behaviour. Inter-observer reliability was tested throughout the training program. The data were reported by the official observers to the SWDA and then to the Swedish Kennel Club.

To remove underrepresented breeds, any breed with n<40 assessments were eliminated from the study. Any breeds for which CI data were not available were also eliminated from the study.

Each test took place along a pathway in a wooded area. A test leader (TL) and the dog’s handler accompanied the dog throughout the assessment. The handler was either the owner of the dog or another person familiar to the dog. The TL gave the handler instructions at every subtest to ensure the test was carried out in a standardised manner. The handler was generally instructed to remain passive so as not to interfere with the dog’s behaviour. There were 10 subtests to the assessment and the TL ensured they were carried out in a standard order; Social contact, Play 1, Chase, Passive situation, Distance-play, Sudden appearance, Metallic noise, Ghosts, Play 2 and, finally, Gunshot. These subtests were set up in advance without the dog or handler present. At each subtest the dog was presented with a situation designed to evoke behavioural responses. An assistant was required for some subtests and this individual remained stationary or out of view until their appearance was required.

An official observer followed the TL and handler to each subtest and recorded the dog’s reaction on a standardised score sheet. The score sheet listed 33 behavioural categories, each divided in intensity from 1 to 5, with 5 being the most intense.

For the complete method of the subtests and score sheet please refer to Svartberg and Forkman (2002).

### Missing Values

The TL or handler could prematurely terminate the assessment. This was necessary if the dog exhibited behavioural distress, became aggressive or was intensely nervous. Some handlers elected to not have their dog exposed to the final test, a gunshot reaction, terminating the assessment after subtest 9. Any dog that did not complete the DMA in full was eliminated from the current study.

## Statistical Analysis

The analysis conducted was a backwards elimination, weighted step-wise regression of each of the 33 DMA variate means on height, weight, CI and sex, and the interactions of sex with height, weight and CI to allow any relationship of the three explanatory variates to change with sexr. We chose this method of step-wise regressions as it is consistent with how factorial experiments are analysed using ANOVA and REML. The frequencies of dogs for each of the averages were used as weights in the regression. The statistical package used was Genstat 17^th^ Edition, (VSN International, Hemel Hempstead, UK). Table 2 provides a summary of all significant explanatory variates.

**Table 2:** Summary of significant p-values and adjusted R^2^ values emerging from stepwise backwards elimination regressions that revealed relationships between Cephalic Index, height, bodyweight, sex and DMA results from 67,368 companion dogs in 33 behavioural variables.

DMA Behavioural Variable	Chosen Behavioural Descriptor	Behavioural Variate	Cephalic Index (CI)	Height	Weight	Sex	CI:Sex	Height: Sex	Weight:Sex	R^2^
**Following 1 (3a)**	Interest in chasing	Chase proneness	< .001	0.002						19.3	
**Grabbing 2 (3b)**	Interest in holding toy	Chase proneness	0.034		< .001					25.8	
**Following 2 (3b)**	Interest in chasing	Chase proneness	< .001		0.004					18.5	
**Grabbing 1 (3a)**	Interest in holding toy	Chase proneness			< .001					24	
**Aggression (8a)**	Defensive toward ghosts	Aggression	< .001	0.045^b^	0.033					28	
**Attention toward assistants (8b)**	Attentive to ghosts	Aggression	< .001		0.013					22.5	
**Aggression (6b)**	Defensive toward dummy	Aggression		0.008^b^	0.02	0.050				6.4	
**Aggression (5b)**	Defensive toward assistant	Aggression			0.017^c^	0.013^d^				11.5	
**Cooperation (1b)**	Cooperation with humans	Sociability	< .001	< .001						39.7	
**Greeting reaction (1a)**	Affectionate toward humans	Sociability	< .001	0.007	0.026^c^					29.4	
**Handling (1c)**	Affectionate toward humans	Sociability	< .001	< .001	0.002^c^	0.050^d^	0.034			34.9	
**Exploration (5c)**	Inquisitive toward assistant	Sociability			0.003	0.010				14.9	
**Contact with assistants (8e)**	Affectionate toward ghosts	Sociability					0.023	0.037	0.049^g^	8.7	
**Play invitation (5e)**	Playfulness with humans	Playfulness & sociability	0.029	0.001		0.017				19.9	
**Grabbing (9b)**	Interest in holding toy	Playfulness			0.006					7.3	
**Grabbing (2b)**	Interest in holding toy	Playfulness			0.011					6.1	
**Interest in play (9a)**	Playfulness with toy	Playfulness			0.025					4.5	
**Tug-of-war (5d)**	Interest in tug-of-war	Playfulness & sociability			< .001	0.003				23	
**Tug-of-war (2c)**	Interest in tug-of-war	Playfulness			< .001	0.029				18.2	
**Interest in play (2a)**	Playfulness with toy	Playfulness				0.046				3.4	
**Startle Reaction (7a)**	Cautious of metallic noise	Curiosity/ Fearlessness	< .001^a^		< .001^c^					32.3	
**Remaining avoidance (7c)**	Prolonged fearfulness of metallic noise	Curiosity/ Fearlessness	< .001^a^		0.034^c^					16	
**Startle reaction (6a)**	Cautious of dummy	Curiosity/ Fearlessness	< .001^a^		< .001^c^	0.007^d^				30.3	
**Exploration (7b)**	Inquisitive toward metallic noise	Curiosity/ Fearlessness			0.010					6.2	
**Exploration (6c)**	Inquisitive toward dummy	Curiosity/ Fearlessness			0.011					6.1	
**Exploration (8d)**	Inquisitive toward ghosts	Curiosity/ Fearlessness					0.012	0.010	0.016^g^	10.7	
**Remaining avoidance (6d)**	Prolonged fearfulness of dummy	Curiosity/ Fearlessness								1.9	
**Remaining approach (7d)**	Prolonged inquisitiveness toward metallic noise		< .001	< .001	< .001^c^	0.027^d^				28.8	
**Activity (4)**	Energetic			0.010	< .001^c^	0.020				21.5	
**Avoidance (10)**	Fearfulness of gunshot				< .001^c^					25.1	
**Interest (5a)**	Inquisitive toward assistant				< .001					14.1	
**Remaining approach (6e)**	Prolonged inquisitiveness toward dummy				< .001	0.011				28.2	
**Avoidance (8c)**	Fearfulness of ghosts						0.032^e^	0.037^f^	0.041	16.1	

^a^ Regression coefficients with CI were positive, apart from those with startle reaction (7a), remaining avoidance (7c) and startle reaction (6a).

^b^ Regression coefficients with height were positive, apart from aggression (8a) and aggression (6b).

^c^ Regression coefficients with weight were positive, apart from aggression (5b), greeting reaction (1a), handling (1c), startle reaction (7a), remaining avoidance (7c), startle reaction (6a), remaining avoidance (7d), activity (4) and avoidance (10).

^d^Regression coefficients with sex were positive, apart from aggression (5b), handling (1c), startle reaction (6a) and remaining approach (7d).

^e^Regression coefficients with CI.sex were positive, apart from avoidance (8c).

^f^Regression coefficients with height.sex were positive, apart from avoidance (8c).

^g^Regression coefficients with weight.sex were positive, apart from contact with assistants (8e) and exploration (8d).

## Results

Thirty-two subtests had at least one significant predictor: height alone (n = 9), bodyweight alone (n = 25), CI alone (n = 13), sex alone (n = 4), height and sex combined (n = 3), weight and sex combined (n = 3) or CI and sex combined (n = 4).

Regression coefficients with CI were positive, apart from both startle reactions (6a & 7a) and 7c remaining avoidance. All regression coefficients with height were positive, apart from two aggression (6b & 8a) behavioural variables. All regression coefficients with weight were positive, apart from 7d remaining approach, both startle reactions (6a & 7a), 7c remaining avoidance, 1a greeting reaction, 1c handling, 4 activity, 10 avoidance and 5b aggression. All regression coefficients with sex were positive, except for 6a startle reaction, 5b aggression, 7d remaining approach and 1c handling. One behavioural variable, 6d remaining avoidance, showed no correlation with CI, height, bodyweight or sex. Regression coefficients with CI and sex combined were positive, with the exception of 8c avoidance. Regression coefficients with height and sex combined were all positive, apart from 8c avoidance. Regression coefficients for weight and sex combined were positive, apart from 8d exploration and 8e contact with assistants.

Correlations among the behavioural variables were derived by factor analysis. The variables were clustered according to the factors described in Svartberg and Forkman (2002). A full description of the method used to calculate the factors is described in Svartberg and Forkman (2002), however, in summary, these factors were created by selecting 25 dogs at random from each breed (47 breeds) with a total of 1,175 dogs from that study. In the current study, our dataset was much larger, with 67,368 dogs. The breeds used to create the factors differ from the ones in our current study. Only 23 of the 47 breeds used in Svartberg and Forkman (2002) are the same as the breeds in the current study. So the alignment between our data and Svartberg and Forkman’s (2002) clusters is not perfect. A general linear regression found some relationship between our data and the clusters devised by Svartberg and Forkman (2002). Height was the only explanatory variate for three of the behavioural variates: chase proneness (p = 0.002), sociability (p = 0.015) and playfulness (p = 0.047). No other relationships were found.

## Discussion

In this study, we report that shorter dogs demonstrate more generalised aggression than taller dogs, while taller dogs show more affection, cooperation and playfulness (with humans). Our results also show that heavier dogs tend to be bolder, more inquisitive and attentive, whereas lighter dogs tend to be more cautious and fearful. These findings align with those of McGreevy et al. (2013), who showed that as height and weight decrease, owners’ reports of undesirable behaviours were more likely. Such behaviours include non-social fear, hyperactivity and attention-seeking behaviours. The current study confirms those links with less desirable behaviours, with data from third parties using the DMA, rather than owners.

The behavioural attributes assessed by the DMA and C-BARQ differ considerably. For example, in contrast to the C-BARQ data that McGreevy et al. (2013) reported upon, the DMA assesses the tendency for dogs to chase a small object rather than chasing a living prey. Our results showed brachycephalic dogs and heavier dogs showed greater interest in chasing and holding prey-like objects. Taller dogs demonstrated more chase proneness in the first chasing test, but this difference disappeared at the second test (the subtest is done twice). Bodyweight was positively correlated with chase proneness, while brachycephalic dogs demonstrated grabbing behaviours on the second test, but not in the first test.

One might predict that chase proneness would be a feature of dolichocephalic dogs given that breeds with this head shape (such as Afghan hounds) have been selected for their ability to hunt and capture prey. Instead, in this study, we found that brachycephalic dogs showed more chase proneness than dolichocephalic dogs. Svartberg et al. (2005) provided a detailed comparison of DMA and C-BARQ data that may help to explain this unexpected result. They showed that chase proneness did not correlate with the ‘chasing’ factor in the C-BARQ questionnaire, but instead with human-directed play interest. So it may be unsurprising that brachycephalic breeds outperformed dolichocephalic breeds in these DMA subtests, as previous studies have proposed that brachycephalic dogs may be more engaged with their owners [17]. As such, dolichocephalic dogs may be less likely to engage in object play, especially with unfamiliar humans.

Aggression is assessed in several DMA subtests rather than one. Aggressiveness has been defined by Svartberg (2007) as a dog’s tendency to both act threateningly, with raised hackles and tail, bared teeth, heightened body posture, growling and to act aggressively, by attacking or biting. Clearly, different motivational states may be encompassed by the catch-all term aggression. Svartberg (2007) suggested that different types of aggressiveness may be directed at different targets. For example, aggression may be directed toward family members, unfamiliar humans, unfamiliar dogs [20], familiar dogs [21], or may be object-related or territorial [22]. It remains unclear whether a general aggressiveness trait exists, and there remains an abiding need for further studies of canine aggression in different situations [22].

There are some subtle but important differences between the DMA subtests that merit discussion. The DMA refer to assistants, ghosts and dummies. The ghosts are assistants dressed in white capes with their faces concealed under buckets bearing a mouth and eyes drawn in black ink. The ghosts make no attempt to gain the dog’s attention, but simply slowly walk toward the dog in increments of 3 steps until they are 4m away, at which point they turn their back on the dog. In comparison, the assistant is dressed in a hooded cape. The assistant’s face is not covered. The assistant uses hand-clapping to catch the dog’s attention and then moves toward the dog in a crouching manner before widening the cape, removing the hood and inviting play by tossing a rag in the air. The dummy is a boiler suit (also known as an overall) that is spread flat on the ground and not visible to the dog until the dog approaches it, at which point it is suddenly pulled up in front of the dog’s path. All dogs are walking with their handlers during this subtest. During the dummy and assistant subtests, dogs are on the lead and handlers remain stationary.

In the current study, dogs were exposed to various different stimuli, objects and unfamiliar humans. The results show that heavier, shorter and brachycephalic dogs demonstrated more aggression toward the ghosts. In addition, heavier dogs showed more attentive staring behaviours toward the ghosts. Meanwhile, heavier, shorter dogs were also more likely to be aggressive in response to the sudden appearance of the dummy. Aggression toward both the ghosts and the dummy–unfamiliar, human-like objects–was therefore inversely correlated with both bodyweight and height.

The current data revealed that lighter dogs demonstrated more aggression toward the assistant. It is difficult to explain why heavier dogs were more aggressive toward ghosts and dummies but lighter dogs were more aggressive toward the assistant. McGreevy et al. (2013) reported that stranger-directed aggression in short dogs correlated inversely with bodyweight. A consideration of short, stocky terrier breeds may help explain this finding as they have been selected for tenacity and a strong instinct to seize and kill prey. McGreevy et al. (2013) also reported that owner-directed aggression was prevalent in shorter dogs. Unfortunately, this relationship cannot be examined using the current study because none of the DMA subtests assess interaction between the dogs and their handlers.

It is possible that the lighter dogs were more fearful of the (unfamiliar) assistants. Lomber and Cornwell (2005) reported that dogs can discriminate their handler from other humans based solely upon face recognition [23]. As a result of their boldness, perhaps, heavier dogs were more aggressive in the face of suddenly appearing objects and disguised humans.

This study also examined the relationship between sex and the DMA data. Our results reveal that male dogs were more likely to be aggressive toward the dummy, while female dogs were more aggressive toward the assistant. The dummy is designed to startle dogs with its sudden appearance, whereas the assistant walks toward the dog in a crouching manner over a longer period and eventually invites play with the dog. It is possible that male dogs felt threatened and instinctively responded to the dummy with defensive aggression, whereas females were more reactive to the slowly encroaching object. This may be similar to a predator approaching a bitch and her litter, the female being defensive of her den [24].

The third factor we considered was sociability. It relates to dogs’ friendliness toward unfamiliar persons [22]. The current data show that taller, brachycephalic dogs were more affectionate, cooperative and interactive with unfamiliar humans. Lightweight, tall and brachycephalic dogs were the most affectionate. This supports previous reports that brachycephalic dogs may be more interactive and affectionate [17]. In terms of sex, female dogs, particularly low-bodyweight females, were especially affectionate when handled. That said, male brachycephalic dogs were also reported as being affectionate.

As bodyweight increased, possessive behaviours became more prevalent. These heavier dogs were more inclined to grab and hold a toy than those with a lower bodyweight. This may reflect hunting and resource guarding responses [25]. Heavier male dogs were especially likely to engage in both of the tug-of-war behavioural variables. As CI increased, possessiveness over a toy was more likely. Perhaps this is because dogs with this morphology are more interactive and engaged with their owners in play [17]. In the current study, dogs with a high CI demonstrated a strong desire to grab and retain a toy when it was offered for the second time, but not for the first time.

To achieve a high score in the greeting reaction and handling subtests, the dog must demonstrate an intense greeting reaction, jumping and whining at the test leader. We found that lighter dogs were the high achievers in these subtests. Arguably, these descriptors are characteristic of an excitable, energetic or hyperactive dog. These findings therefore complement those of McGreevy et al. (2013), who reported that lighter dogs were especially likely to be excitable, energetic and hyperactive. This apparent alignment demands confirmation and, as such, emphasises the need for a validated canine ethogram that eliminates any chance that canine responses to tests remain open to interpretation. Overall (2014) has recently called for collaboration on a global project to produce and agree upon a standardised set of behavioural descriptions [26].

Distinct from sociability, playfulness in the DMA describes the dog’s tendency to run after a thrown rag, grab it and to then play tug-of-war [22] and reports results from two subtests (Tug-of-war (5d) and Play invitation (5e)).

Brachycephalic, tall, male dogs were both more playful and sociable with humans. This raises interesting questions about whether we are deliberately selecting large, boisterous and playful dogs as companions or whether sociability and boldness during play are inadvertently selected when we breed large dogs. Heavier dogs and male dogs demonstrated more intense play behaviour in a tug-of-war situation with tugs and twitches, even when the assistant was passive, until the assistant lets go of the object. Tug-of-war is a game of possession, a test of strength and an opportunity for social engagement. Over time, we may have selected dogs that would enjoy tug-of-war, but at the same time also deliver a hard, sustained bite. Several authors have reported that male dogs are bolder than females [18, 27]. The social rank of a dog can be estimated from its ability to gain and retain access to valuable resources, although any such tests must be qualified by specifying the resource in question. The ability of a dog to retain a given resource and displace others from it can be learned and so contribute to the development of social order [28]. Such resources may include tug-of-war objects. Scott and Fuller (1965) studied puppies contesting a resource and found that males tended to win against females [29]. The current study suggests that males demonstrate more interest in possession games. However, given that they are generally heavier than females, this factor must be considered before one concludes that they are socially dominant, as has been proposed [30].

The playfulness factor focuses on results from both Play subtests, subtest 1 and subtest 9. Heavier dogs were the most likely to grab and hold a toy and demonstrated most interest in object play. This may, at least in part, be a learned response in that, due to their weight, these dogs tend to win games of possession whereas lighter dogs may be less successful. Heavier dogs have also been selected for fighting and restraining behaviours. The findings of Rooney and Bradshaw (2001) support this, as dogs in that study were more motivated during play sessions in which they were allowed to win, than those in which they lost [31].

Of all the factors considered in the current DMA analysis, fearfulness is among the most studied traits in dogs [22]. It is associated with avoidance and flight behaviours, low body posture, low tail and ears, trembling, salivation and vocalisation [22].

The current study shows that lighter, dolichocephalic dogs were the most cautious of metallic noise and showed prolonged fearfulness. Lighter, dolichocephalic female dogs were the most cautious and startled by a suddenly appearing dummy. If the dummy is considered realistic enough to represent an unfamiliar human, these results align with those of McGreevy et al. (2013), who showed that stranger-directed fear was most common in dolichocephalic dogs. In considering why dolichocephalic breeds may be more fearful, it is worth noting differences in visual acuity that are associated with skull shape. In contrast to brachycephalic dogs, it is believed that dolichocephalic dogs have poorer visual clarity centrally [7, 11] and therefore may be less able to visually and cognitively process a suddenly appearing dummy directly in their path. It is also possible that these dogs are more vulnerable to attack and therefore demonstrate avoidance and flight behaviours to avoid injury.

In the current study, inquisitiveness depended on the stimulus in question. Dogs that showed interest in exploring the dummy or the source of a metallic noise were heavier dogs. Meanwhile, dogs that were inquisitive toward the ghosts were brachycephalic males, tall males and lighter females. These morphological attributes are identical to those of the dogs that scored highly on the playfulness and sociability factors. It is worth considering whether some dogs may be optimistic about the potential for a stimulus to be socially satisfying. The ghost is likely to be a foreign stimulus not previously encountered. More-social dogs may assume the ghost to be a social object, or these dogs may have learned that exploring ambiguous stimuli can be rewarding, resulting in a greeting or game of tug-of-war.

Attachment and fear were associated with shortness in the dataset reported by McGreevy et al. (2013). In the current study, we report only one association with height. Taller male dogs were more inquisitive toward the ghost. This inquisitiveness could be considered the opposite of fear and safe-haven attachment [32] as the dogs demonstrate interest and travel away from the handlers to do so.

Six subtests were not assigned to a factor, but the results from these merit discussion. Brachycephalic, tall females with a low bodyweight were more inquisitive toward a metallic noise. Interestingly, these low-bodyweight dogs were the most fearful of the gunshot. This is difficult to explain, although Svartberg (2007) suggested that narrow sub-types of fearfulness may exist and that, as such, fearfulness of one stimulus may not be associated with fear of another stimulus.

Dolichocephalic females, short females and heavy male dogs demonstrated fearfulness of the ghosts. We have reported that dolichocephalic and short dogs have a greater tendency to show fear. However, we have also reported that heavy male dogs tended to be bold and inquisitive. Here we have found heavy males to be fearful of the ghosts. There was no correlation with CI, which suggests visual differences were not responsible for this result. Again, it is possible that these dogs demonstrate distinct fear responses to specific stimuli.

Heavy dogs were the most inquisitive toward the assistant, and heavy males demonstrated prolonged inquisitiveness toward the dummy. These findings are supported by our results on evidence of curiosity.

One variable reports directly on the dog’s physical activity, while the handler stands passively. For this variable, entitled Activity, lighter dogs were reported as being energetic and frequently changing their activity during the rest period. These findings support those of McGreevy et al. (2013), that lighter dogs tended to be more excitable, energetic and hyperactive. In the current analysis, lighter dogs were also found to be the most cautious and fearful.

### Conclusion

The current study demonstrates that morphology and behaviour are intricately linked in the domestic dog. Its findings support previously reported relationships between bodyweight, height, CI and behaviour. In the current study, we have built upon these foundations with the added variable of sex, showing that the covariance of morphology and some behaviours depends on sex. There is still a need for further investigation into the mechanisms that underpin covariance with morphology, to reveal the relative roles of genetic selection or environmental adaptation in the emergence of these relationships.

## Supporting Information

S1 Dataset Height, Bodyweight and Cephalic Index data. (XLSX)
